# Supplementary material for: A fast lasso-based method for inferring higher-order interactions
Source: PLoS Comput Biol. 2022 Dec 29;18(12):e1010730. doi: 10.1371/journal.pcbi.1010730 (PMC9833600; doi:10.1371/journal.pcbi.1010730)
Supplement: S1 Appendix — (PDF) [file pcbi.1010730.s001.pdf]

S1 Appendix

We can considerably reduce the size of the active set by compressing the columns. Since we have a sequence of increasing integers we can store only the offset from the previous entry, keeping the entries small. The resulting sequence of (mostly) small numbers can then be efficiently stored using integer compression methods. We describe the compression method we use in Section 1 and compare it to other methods in Section 2.

1 Simple-8b

Simple-8b is a non-SIMD compression scheme, with performance comparable to other state of the art methods [1–3]. While SIMD-based compression schemes can often offer significantly improved compression and decompression speed [1, 4], their implementation is architecture dependant. Simple-8b only requires a CPU be able to efficiently handle 64-bit arithmetic, and does not significantly underperform compared to state-of-the-art SIMD techniques in our testing (Section 2).

Simple-8b is a 64-bit variation of the Simple-9 encoding scheme [5], and stores a sequence of integers in a single 64-bit word. The number of integers stored depends on the size of the largest one, and is indicated by a four bit 'selector'. The remaining 60 bits are divided into integers of size $1, 2, 3, 4, 5, 6, 7, 8, 10, 12, 15, 20, 30$, or 60, with between 240 (only possible if all values are zero) and one integer stored. As seen in S1 Fig, this considerably reduces the size of $\mathbf{X}_2$ in our test data (two sets from [6], one with $p = 100,\ n = 1,000$, another with $p = 1,000,\ n = 10,000$). In the larger $p = 1,000$ set, total memory use is reduced by over 85% compared to storing integers directly. It is worth noting that this compression works well even for non-sparse sections of the matrix, since the offsets are extremely small. In an extreme case, we can store up to 240 sequential 1's in a single 64-bit word.

2 Comparing Methods

While Simple-8b allows our implementation to be used on any 64-bit CPU, we could also take advantage of SIMD-based methods where such CPU instructions are available. To determine whether this is a worthwhile improvement, we compare our Simple-8b implementation to a number of state of the art alternatives.

Recent work suggests TurboPFor [7] has a particularly high compression ratio [3]. We therefore compare the best performing methods from TurboPFor against our implementation of Simple-8b (S2 Fig). The tests are performed using an eight-core (16 SMT threads) Intel Xeon Gold 6244 CPU. To compare these methods, we perform 50 regression iterations on a test data set of $p = 1,000$ genes and $n = 10,000$ siRNAs. We examine the total time taken for the process, as well as the total memory used and time for the regression function alone (excluding calculating and compressing the interaction matrix).

We see that both the time to produce the compressed matrix (seen in S2 Fig as the difference between total time and lasso-only time), and the running time are comparable for all TurboPFor methods. The compression time is not comparable for all methods. Our Simple-8b implementation compresses columns in parallel, whereas TurboPFor does not. Columns are decompressed in parallel in both cases. While every TurboPFor method we tested improved the compression ratio compared to Simple-8b (S2 Fig A), we consistently found that the running time was longer (S2 Fig B). It is possible that this is a result of the way the columns are being read in each method. Using TurboPFor, we compress and decompress entire columns at a time. With our Simple-8b implementation, we process each 64-bit word separately. This allows us to use the column as it is being decompressed. Avoiding re-reading the column after decompression also allows the entries to be evicted from the cache earlier.

While it is also possible to process compressed words as they are read using the tested TurboPFor methods, there does not appear to be a significant difference in compression that would justify doing so.

References

1. Schlegel B, Gemulla R, Lehner W. Fast Integer Compression Using SIMD Instructions. In: Proceedings of the Sixth International Workshop on Data Management on New Hardware - DaMoN '10. Indianapolis, Indiana: ACM Press; 2010. p. 34–40.

2. Mallia A, Siedlaczek M, Suel T. An Experimental Study of Index Compression and DAAT Query Processing Methods. In: Azzopardi L, Stein B, Fuhr N, Mayr P, Hauff C, Hiemstra D, editors. Advances in Information Retrieval. Lecture Notes in Computer Science. Springer International Publishing; 2019. p. 353–368.

3. Trotman A, Lin J. In Vacuo and In Situ Evaluation of SIMD Codecs. In: Proceedings of the 21st Australasian Document Computing Symposium. ADCS '16. Caulfield, VIC, Australia: Association for Computing Machinery; 2016. p. 1–8.

4. Lemire D, Boytsov L. Decoding Billions of Integers per Second through Vectorization. Software: Practice and Experience. 2015;45(1):1–29. doi:10.1002/spe.2203.

5. Anh VN, Moffat A. Inverted Index Compression Using Word-Aligned Binary Codes. Information Retrieval. 2005;8(1):151–166. doi:10.1023/B:INRT.0000048490.99518.5c.

6. Elmes K, Schmich F, Szczurek E, Jenkins J, Beerenwinkel N, Gavryushkin A. Learning Epistatic Gene Interactions from Perturbation Screens. PLOS ONE. 2021;16(7):e0254491. doi:10.1371/journal.pone.0254491.

7. powturbo. Powturbo/TurboPFor-Integer-Compression; 2020.
