# Supplementary material for: A fast lasso-based method for inferring higher-order interactions
Source: PLoS Comput Biol. 2022 Dec 29;18(12):e1010730. doi: 10.1371/journal.pcbi.1010730 (PMC9833600; doi:10.1371/journal.pcbi.1010730)
Supplement: S1 Table — (PDF) [file pcbi.1010730.s002.pdf]

Table 1. Predicted top 50 SNV effects.

Lasso Estimate	Least Squares Estimate	pval	SNV 1	SNV 2
-0.024	-1.76	0.01	813809 sub. T C	—
-0.013	-5.02	0.02	3789873 sub. T C	—
-0.039	-1.68	0.02	4617770 sub. C T	—
-0.007	9.71	0.03	5111434 sub. A G	—
-0.006	-2.83	0.03	4153211 sub. A G	5719820 sub. A G
0.000	-1.15	0.05	6081497 sub. T C	—
-0.007	-2.25	0.06	4271404 sub. T C	—
-0.023	-2.25	0.06	63407 sub. T C	—
-0.063	-2.25	0.06	184347 sub. T C	—
-0.001	-12.62	0.07	137771 sub. A G	—
-0.008	-2.76	0.10	3654726 sub. T C	3869317 sub. A G
-0.019	-1.03	0.11	1886446 sub. T C	—
-0.045	13.18	0.11	442184 sub. C G	—
-0.001	-2.10	0.12	986363 sub. A G	—
-0.003	-1.84	0.13	2996864 sub. C T	3869317 sub. A G
-0.030	-1.60	0.16	1810265 sub. A C	—
-0.012	0.76	0.20	2033361 sub. A C	—
-0.009	-7.03	0.22	3318136 sub. T C	—
-0.038	8.19	0.23	176977 sub. A G	—
-0.002	2.44	0.24	172758 sub. T C	—
-0.009	2.00	0.24	4003269 sub. T C	—
-0.034	8.48	0.24	4153211 sub. A G	—
-0.008	-6.99	0.28	3419855 sub. T A	4153211 sub. A G
-0.031	-1.25	0.30	4847782 sub. A C	—
-0.005	-1.25	0.30	1383522 sub. A G	—
-0.005	-1.25	0.30	176977 sub. A G	5133603 sub. A G
-0.004	2.36	0.30	3419855 sub. T A	4920968 sub. C A
-0.040	2.44	0.31	3944454 sub. T A	—
-0.004	-0.84	0.40	3195104 sub. T C	—
-0.028	-0.42	0.40	2211528 sub. T G	—
-0.013	2.14	0.51	313283 sub. A C	—
0.000	0.90	0.51	3601029 sub. T C	3869317 sub. A G
-0.005	4.38	0.53	3419855 sub. T A	—
-0.010	2.64	0.55	5254860 sub. T C	—
-0.016	-0.31	0.69	2093685 sub. A T	—
-0.007	1.02	0.73	3869317 sub. A G	4687756 sub. A G
-0.044	-0.59	0.78	4453098 sub. A G	—
-0.005	0.24	0.80	3563823 sub. A G	—
-0.039	1.21	0.81	4125422 sub. T C	—
-0.002	0.57	0.88	3869317 sub. A G	4966957 sub. C T
-0.103	0.21	0.95	3869317 sub. A G	—
-0.005	—	1.00	956627 sub. A G	—
-0.001	—	1.00	5856921 sub. A G	—
-0.002	—	1.00	3869317 sub. A G	4617770 sub. C T
-0.014	—	1.00	3869317 sub. A G	4847782 sub. A C
-0.008	—	1.00	4153211 sub. A G	4453098 sub. A G
-0.046	—	1.00	3869317 sub. A G	4453098 sub. A G
-0.006	—	1.00	4153211 sub. A G	5990479 sub. C G
-0.009	—	1.00	3195104 sub. T C	4153211 sub. A G
-0.046	—	1.00	986363 sub. A G	3869317 sub. A G
