# Supplementary figures and images for: A fast lasso-based method for inferring higher-order interactions
Source: PLoS Comput Biol. 2022 Dec 29;18(12):e1010730. doi: 10.1371/journal.pcbi.1010730 (PMC9833600; doi:10.1371/journal.pcbi.1010730)

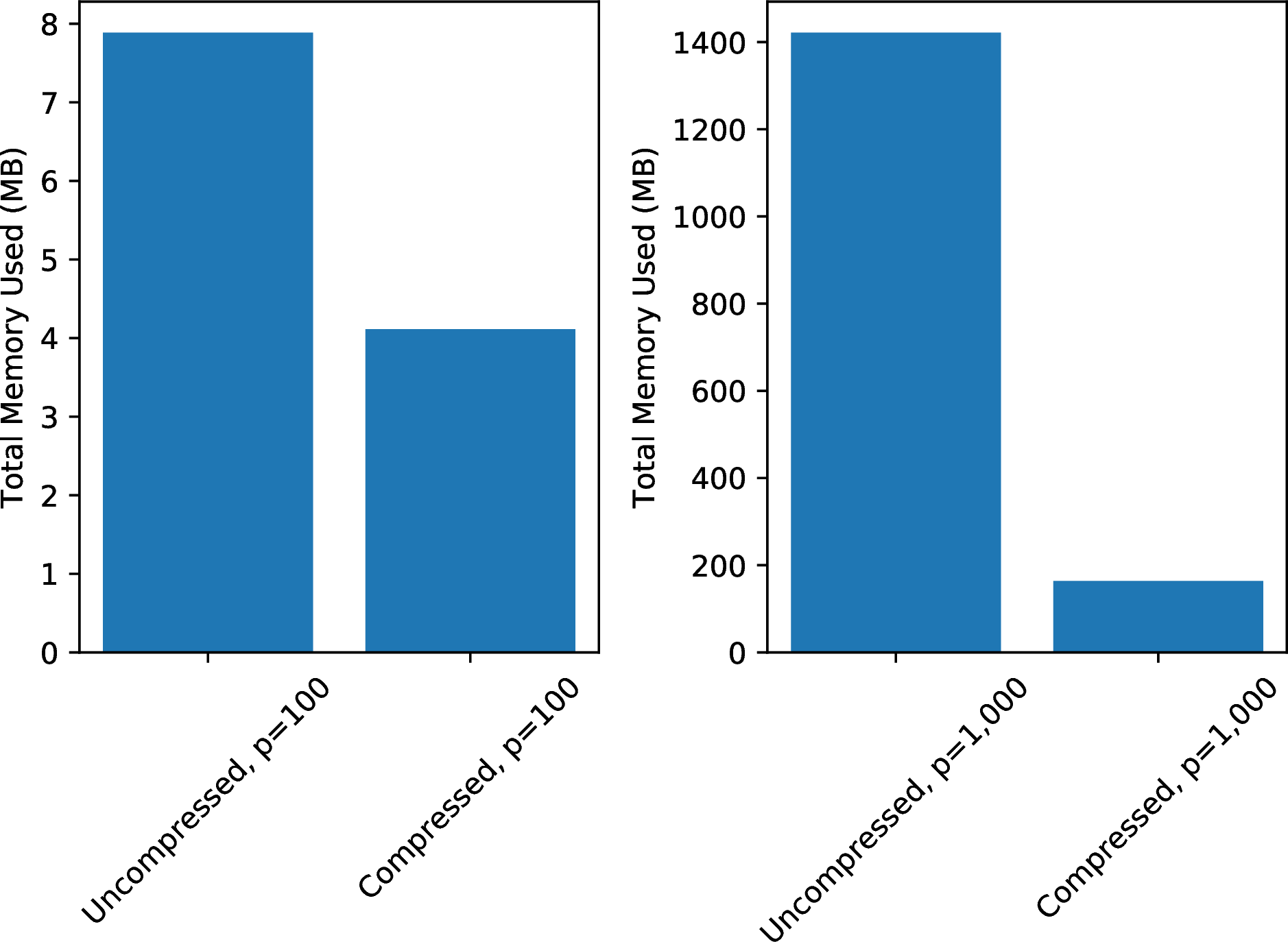

Supplement: S1 Fig — Note that this is the total peak memory use of the program, not solely the memory used by the matrix X2. In both cases n = 10 ⋅ p. (TIF) [file pcbi.1010730.s003.tif]

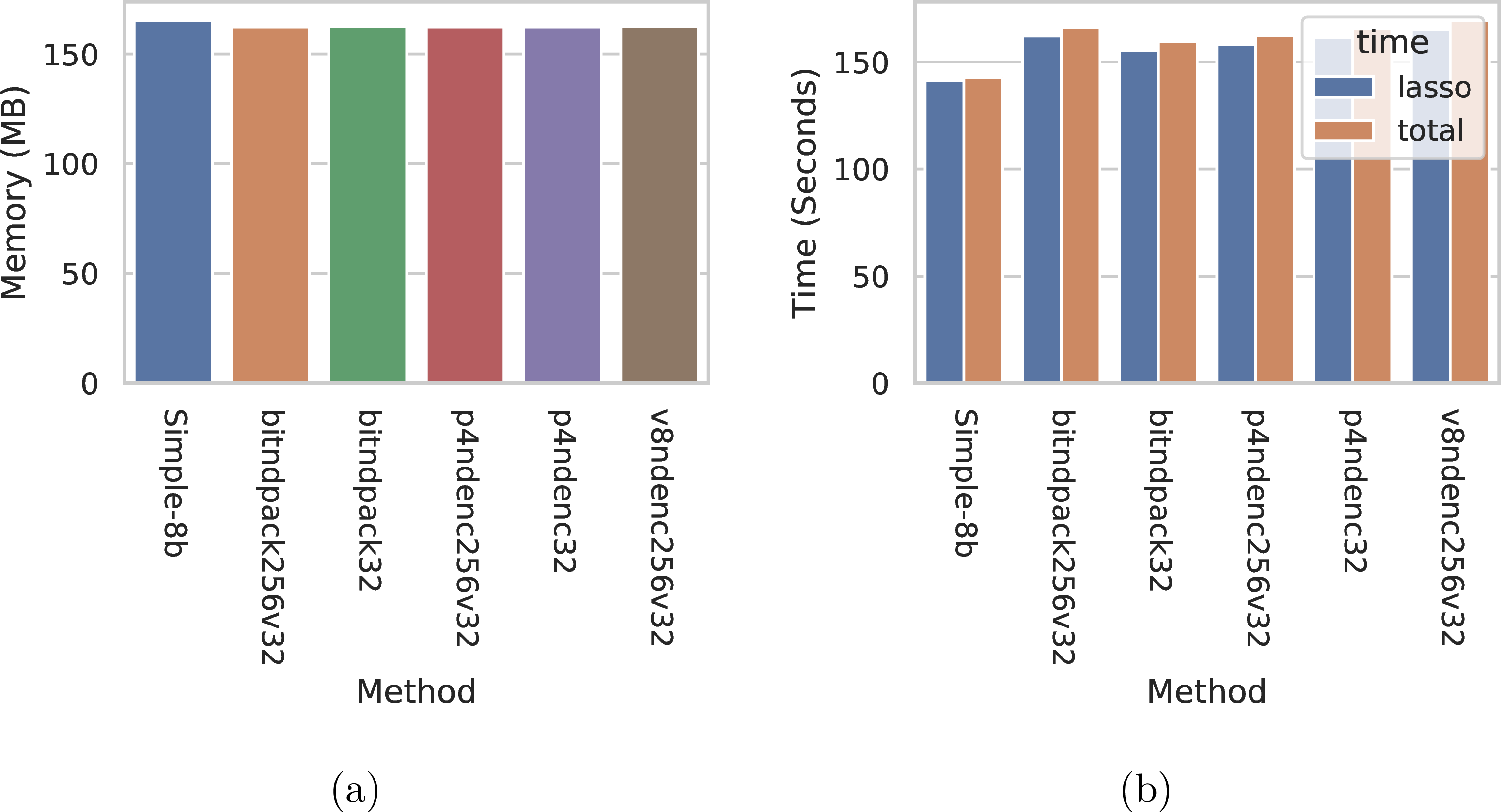

Supplement: S2 Fig — (a) Total memory used, compressing the sparse X2 matrix with each method. (b) Total time taken and time taken (including compressing X2) and time taken for lasso regression alone, using each method. (TIF) [file pcbi.1010730.s004.tif]
