# Supplementary material for: A fast lasso-based method for inferring higher-order interactions
Source: PLoS Comput Biol. 2022 Dec 29;18(12):e1010730. doi: 10.1371/journal.pcbi.1010730 (PMC9833600; doi:10.1371/journal.pcbi.1010730)
Supplement: Attachment — Submitted filename: reply.pdf [file pcbi.1010730.s005.pdf]

Response to Reviewers

August 21, 2022

We would like to thank the editor and both reviewers for their helpful suggestions. Some of these had already been implemented since the initial submission, and many of the rest have now been addressed in the paper or package. In particular, the ability to include non-binary values in the input matrix X has been implemented. There are two other additions, the option to identify and exclude identical effects (now described in section 2.8), and an option to ignore interactions between features that have no main effect (section 2.7). These were originally included in the discussion of potential future work. As well as the changes to the paper and package, we address each comment individually in the itemised list below.

1 Reviewer #1

- I was able to install the package and run:

```
> output <- interaction_lasso(X, Y, n = dim(X)[1], p = dim(X)[2],
    lambda_min = -1, frac_overlap_allowed = -1, halt_error_diff
    =1.01, max_interaction_distance=-1, use_adaptive_calibration=
    FALSE, max_nz_beta=-1, max_lambdas=200, verbose=FALSE,
    log_filename="regression.log", depth=2, log_level="none",
    estimate_unbiased=FALSE, use_intercept=TRUE)
Error in interaction_lasso(X, Y, n = dim(X)[1], p = dim(X)[2],
    lambda_min = -1, :
unused arguments (frac_overlap_allowed = -1,
    use_adaptive_calibration = FALSE)
> output <- interaction_lasso(X, Y, n = dim(X)[1], p = dim(X)[2],
    lambda_min = -1, halt_error_diff=1.01, max_interaction_distance
    =-1, max_nz_beta=-1, max_lambdas=200, verbose=FALSE,
    log_filename="regression.log", depth=2, log_level="none",
    estimate_unbiased=FALSE, use_intercept=TRUE)
total entries: 49944
```

  This should obviously be fixed in the readme of the package.
  – *This was for an old version, the readme has now been updated.*

- `github.com/bioDS/lasso_data_processing` leads to a 404.
  – *The repository is now public.*

- The authors should at least address the sizes of the data sets directly in their manuscript's main part. This is important information and should be readily available to the reader. If I understand correctly, the sizes are nxp = 1000x100 and 10000x1000. That seems relatively small compared to the real data sets with dimensions 6703x19533 and 259x174334. The authors should explain why the relation of n and p in the real data sets is inverse to the simulated ones. In the discussion the authors mention /approx 67000 siRNAs and the n=6703 from before could be a mistake. The authors address the limitation, where they talk about data set size and three-way effects, i.e., when their method breaks down. Why is that not directly shown in the simulations?
  - *The simulations have been described in more detail, it should no longer be necessary to read Elmes et al. 2021 to understand them.*
  - *The simulation scenario where $n \gg p$ is the ideal case, and we aim to demonstrate that the method works here. We have replaced the large set with one where $p \gg n$, to demonstrate the method under less ideal circumstances.*
  - *Accuracy of three-way effects has been discussed on more detail.*

- In the discussion the authors mention /approx 67000 siRNAs and the n=6703 from before could be a mistake.
  – $67,000$ *was the mistake, this has been corrected.*

- The authors should add more simulations to roughly show when this three-way effect identification becomes infeasible.
  – *The original paper simulated a specific fraction of all possible effects in the three-way case. This meant that there were tens of thousands of true positives, and recall was essentially zero. We have replaced this with a small number of both pairwise and three-way effects, and re-run the simulations. In these simulations we find true positive three-way effects are extremely rare. While almost all predictions are false positives, we show a significant fraction of these are indistinguishable from some true positive effect. This is now discussed in section 3.2.*

- The authors need to explain why such low precision and relatively high (only for small data sets) recall is favored in their performance. Are false positives considered the lesser evil?
  – *Effect strength and the number of effects can be used to choose between low precision with high recall, and high precision with low recall. This has been described in section 3.1.1. In summary, yes for the applications we had in mind the main objective was to identify true positives.*

- The authors should consider the area under the curve for the ranked effects, i.e., precision and recall for the first (i.e., strongest) effect that is found, then for the first and second, then for the first, second and third effect, and so on.
  – *This has been added in section 3.1.1.*

- Additionally, the authors never show recall for the 3-way effects. The question is, how relevant is this with a precision of 6%? How good is random in this case? I.e., what is the prevalence of the effects in the data sets?
  – *Overall precision and recall are near zero for three-way effects, however the strongest predictions are relatively precise. This is now discussed in section 3.2. The three-way simulated data sets are now discussed in more detail (including the number of effects present) in section 2.10.1.*

- If I read [15] correctly, the data sets include only {5, 20, 50, 100} + {0, 20, 50, 100} main and pairwise effects. What about three-way effects in the simulated data? That is of course never talked about in [15] but also not in the main manuscript. Hence, the referral to the simulated data in [15] makes no sense for three-way effects.
  – *We have replace the simulations from [15] with ones described fully in this paper, including simulations with three-way effects (section 2.10.1). (This is largely to remove the strong hierarchy simulation from [15], which we do not need to assume here.)*

- Just to be clear, the authors never use the actual effect size for performance evaluation. The main concern is just to identify true positives, correct? Maybe they should make that a bit clearer in the text.
  – *This has been clarified in the text.*

- As I understand, the authors look for cell growth and survival genes in their list of identified effects from the infectx data. While the top found genes seem to certainly make sense and are a nice result in itself, I suggest if possible an additional gene set enrichment analysis to make this part more systematic and quantifiable.
  – *This would require a way to sort genes according to their effect on cell growth or survival. We do not have any measurements of gene expression to associate with cell survival. While we do know siRNA targets, and have predictions of off-targets, we do not have measurements of the effect of silencing individual genes. As a result, there is no clear way to sort the genes according to their likely contribution to cell survival, making gene set enrichment analysis difficult.*

- "... so the possibility of a true combined effect of Ciprofloxacin resistance cannot be ruled out... ", but seems highly unlikely given the simulation results. Also, even if the variants were characterized as not affecting Ciprofloxacin resistance, even then a true combined effect could still not be ruled out. Hence, this last part of the sentence seems like a NULL statement and should be removed.
  – *This sentence has been removed.*

- I noticed several long sentences like this one and suggest in general to keep sentence length as concise as possible..

– Unnecessarily long sentences in the antibiotic section have been broken up.

- "Additional genes identified using our method...". Did you use any other methods to identify genes in this section? If so, this is not clear at all.
  – This has been re-worded to clarify that these were only more of the effects found with Pint.

- The authors again make the argument in the discussion that tools need to be tailored towards larger data sets and higher order interactions. That is why they developed Pint. However, if accuracy is so low for relatively small data sets (where speed is not the issue), how can they make any prediction on even larger data sets? Accuracy will drop even more. Like mentioned above maybe the top hits still look very good and an AUC as accuracy measure might visualize that in the simulations.
  – We have added the suggested AUC plot, and it appears that strong predictions are largely correct, even when overall precision is very low.

- The authors mix notation, which can be confusing. E.g., /phi is used once to denote the probability density function of the normal distribution and another time as "r, the last time column x was included in the working set" (p.5). This type of ambiguity should be avoided.
  – ϕ *has been replaced with* ρ *to avoid ambiguity.*

- It is also confusing reading about column X_x and column x, which is also referred to as the index ("... interaction with x ...", this time x is the column? p.5). The authors should make sure that their notation is consistent and unambiguous throughout the whole manuscript.
  – We now consistently refer to column X_x *rather than 'column x'.*

- Also defining $/phi_x = r$ and then using `r/cdot/phi_x` in an equation seems strange. It would be less confusing just defining `/phi_x` as "the residuals, the last time column x was inc..." without misusing r. It seems that the residuals are used on page 4 eq.4 for the first time. Even though it is a common notation in the field, they still need to be formally introduced.
  – We now use this definition. Residuals are now defined in equation 4.

- Off-target effects are prediction using RIsearch2 → Off-target effects are predicted using RIsearch2
  – This has been corrected.

- In the follow up sentence: the gene should be called the "off-target". The respective off-target effect would be the inhibition of the gene's expression.
  – Sentence has been clarified.

- "... further increasing the number of threads has to noticeable impact on performance..." → "... further increasing the number of threads has no

noticeable impact on performance..."
– This has been corrected.

- The "Method" in figure 4 is supposed to be Pint, right? Please correct that/make that clear.
– Yes, this has been clarified in axis label and caption (now figure 6)

- The reference of [38] seems to be incomplete.
– This has been corrected.

- "We can considerably reduce the size of the active set by compression the columns." → "We can considerably reduce the size of the active set by compressing the columns."
– This has been corrected.

2 Reviewer #2

- In Figure 4c, it's noted that the precision for the 3way interaction terms was extremely low. In Section 3.2 the authors noted that this was mostly due to overlapping three-way effects? Could authors sinclarify what they meant by "overlapping three-way effects"? columns are identical? If so, how many False positives due to such overlapping three-way effects? When n=1000 in the binary setting, the interaction term is either 0 and 1. How many such three way interaction terms were detected significant? Such low precision may limits the usefulness of the method in such applications, which may deserve some discussion
– Overlapping effects (identical columns) are now more clearly described in the text. The number of false positives due to this has also been investigated and is discussed.

- Can Pint apply to non-binary data? I can see many applications may go beyond the SNP type of variables, (ie. continuous instead of binary). But the binary setting of variables may greatly limit the usage of this method. For example, in gene-gene interaction case, all expression may be continuous. The overlapping-effect presented in Figure 4C maybe not an issue. If Pint works for such data, it will expand the scope of applications. Can authors discuss it?
– When this paper was originally submitted, this was not possible. It has been added since then, and is now discussed in section 2.9.

- On page 7, section 2.1 should $X \in \{0,1\}^p$ be $X \in \{0,1\}^{nxp}$?
– It should, this has been corrected.
