# Supplementary material for: A fast lasso-based method for inferring higher-order interactions
Source: PLoS Comput Biol. 2022 Dec 29;18(12):e1010730. doi: 10.1371/journal.pcbi.1010730 (PMC9833600; doi:10.1371/journal.pcbi.1010730)
Supplement: Attachment — Submitted filename: response_ploscb_v2.pdf [file pcbi.1010730.s006.pdf]

# Response to Reviewers

October 24, 2022

We would like to thank the reviewer for clarifying both of these points, as well as pointing out a number of minor erors in the revised paper. We have now made the recommended changes. Replacing the precision/recall analysis with ROC plots involved re-running most of the simulation analysis (since we previously collected only summaries of the results) and we have changed a number of the parameters to produce a clearer comparison in the ROC plots. In particular, we significantly increased the maximum number of effects, since the previous low limit resulted in a difficult to interpret plot with very few true positives. As a result, the overall running times have also changed.

We include below a point-by-point summary of the changes made to address each comment.

1. Maybe the authors misunderstood my previous concerns regarding the precision/recall plots and my suggestion to replace (not add) them with AUC plots. Precision and recall in the single digits do not show that a method is working reasonably well (on the contrary). However, precision and recall are also much dependent on the cutoff for the output values (in this case !=0 and ==0, correct?). Considering a small effect only marginally larger than zero as an effect and a large effect far larger than zero as the exact same effect seems unreasonable. The AUC does not depend on such a cutoff. Hence, I strongly suggest that the authors replace precision/recall plots with AUC plots unless there is a valid reason not to do so (which I can not see). The single shown AUC plot seems to show that the method works very well (is there a corresponding precision/recall plot to it?, because I have a hard time believing that this AUC plot would result in low recall/precision numbers or is this simulation run an outlier? There is no error shown.). Especially, since the authors let their method stop after some (arbitrary? At least in the application to real data. How is that number determined?) number of found non-zero effects, the AUC shows that the top effects should be true positives.
   – *The precision/recall plots have now been replaced with AUC plots. We previously only used the ROC to show that among non-zero predictions, strength is a good indication of accuracy. The effects that regularisation excluded (i.e. that were zero) were left out. The AUC was very high when we only consider these effects (this is now compared in figure 6 for*

*all methods). We stopped the method after a relatively small number of effects because these are likely to be the correct predictions. Our intention in the real data analysis is only to find a small number of true effects, rather than to model everything.*

2. I do not follow the explanation of why an enrichment analysis is not possible. You have your identified genes and there are databases (e.g., http://www.gsea-msigdb.org/gsea/msigdb/index.jsp) with known biological processes and other common functional gene groups available. Based on this you could for example compute a simple confusion matrix (overlap of your genes and the group, exclusive to the group/your genes, and rest of the gene population) and use Fisher's exact test (or equivalent) to try to quantify the overlap. There is no need to rank the results.
   *– We misunderstood the original request, (my understanding is that the method from Subremanian et al. 2005 would be difficult to apply here). We have now included a gene-set analysis using the curated collection from gsea-msigdb in section 3.3.*

1. I assume the authors actually simulated each data set several times to get the variance of the accuracy plots. Is that and how often this is done stated anywhere?
   *– We simulated each set either 10 or 50 times, this has been clarified in section 2.10.1*

2. "... We prepared one simulated and two experimental data sets ..." Should be nine (without repetition for random noise) simulated data sets, right?
   *– There were actually 70 simulations in total, this is now specified in the text*

3. Response: "In summary, yes for the applications we had in mind the main objective was to identify true positives. " But this objective is not fulfilled, if you identify a lot of false positives due to low precision. In a real case you cannot distinguish true and false positives. I fail to see the motivation here.
   *– Our goal is not to find all true positives, but to provide a method for finding some likely candidates. It is enough that the top effects (sorted by strength) are mostly true positives (as we see in the roc plots). We can then say that while we do not know every cause of a particular phenotype, these are the most likely candidates for further investigation.*

4. "... we maintain a vector Vk of the values for each column of the input matrix Xk." => "... we maintain a vector Vk of the values for each column Xk of the input matrix X."
   *–This has been corrected.*

5. "datasets" => "data sets" (or be consistent with datasets).
   *–This has been corrected.*

6. “To begin with, we take simulated a simulated matrix ...” => “To begin with, we simulated a matrix ...”
   –*This has been corrected.*
